# Supplementary material for: Differential activation of a frontoparietal network explains population-level differences in statistical learning from speech
Source: PLoS Biol. 2022 Jul 6;20(7):e3001712. doi: 10.1371/journal.pbio.3001712 (PMC9292101; doi:10.1371/journal.pbio.3001712)
Supplement: S1 Fig — (A) SSS test outcome. Histogram of 388 PLVs obtained in a previous work1 with 2 different versions of the SSS test. Black dots represent the participants selected to complete the fMRI protocol. Black line represents the threshold value adopted in this work to separate high and low synchronizers: PLVthreshold = 0.49. A k-means clustering algorithm using a squared Euclidean distance metric was applied over this distribution (N = 388). The threshold value is the midpoint between the 2 clusters’ centers. (B) Scatterplot displaying participants’ PLV during AS inside the scanner as a function of the PLV from the SSS test. Red line represents the correlation of the data. The correlation is displayed for visualization purposes, to emphasize that the synchronization of low synchronizers is consistently worse than that of highs during the AS block. The correlation within groups remains significant only for high synchronizers (rHIGH = 0.45 pHIGH = 0.044; rLOW = 0.21 pLOW = 0.31). (C) Percentage of correct responses for the statistical word learning task during PL and AS conditions inside the scanner on the entire sample. (D) Percentage of correct responses for the statistical word learning task during PL and AS conditions inside the scanner for the low (blue color) and the high (orange color) synchronizers. The mixed-model analysis of this dataset yielded a significant difference between conditions (main effect of Condition (PL > AS), χ2 = 5.40, p < 0.05)), and a main effect of group close to significance (Highs > Lows; χ2 = 3.67, p = 0.055), and a trending Condition*Group interaction (χ2 = 2.74, p = 0.098). Dots: model predicted group means. Bars: 95% confidence interval. Data for S1A and S1B Fig can be found in S5 Data. Data for S1C and S1D Fig can be found in S6 Data. AS, articulatory suppression; PL, passive listening; PLV, phase locking value; SSS test, Spontaneous Speech Synchronization test. (DOCX) [file pbio.3001712.s001.docx]

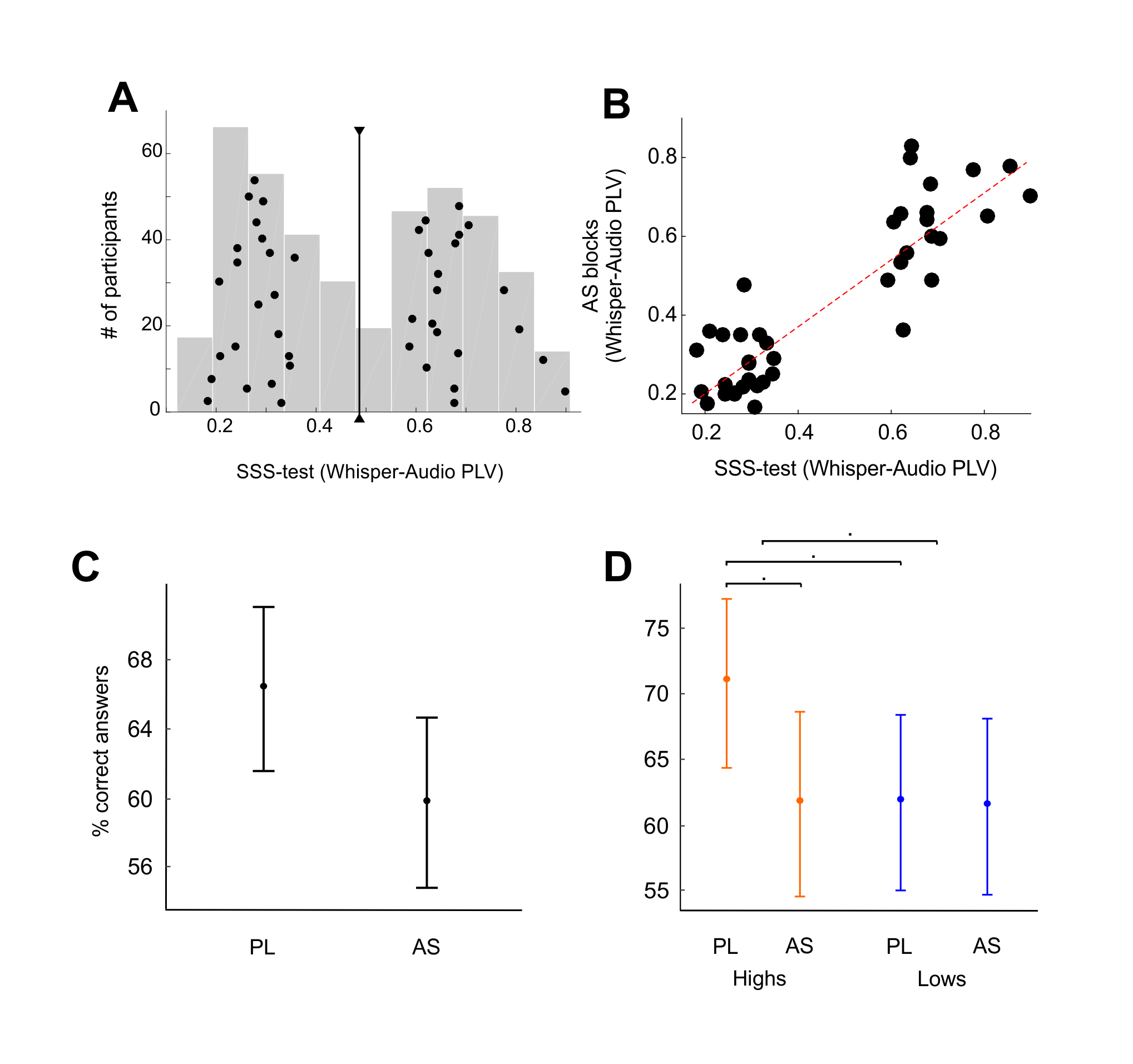


**S1 Fig. Behavioral performance in the scanner. (A)** SSS-test outcome. Histogram of 388 PLVs obtained in a previous work^1^ with two different versions of the SSS-test. Black dots represent the participants selected to complete the fMRI protocol. Black line represents the threshold value adopted in this work to separate high and low synchronizers: PLV_threshold_ = 0.49. A *k-means* clustering algorithm using a squared Euclidean distance metric was applied over this distribution (N = 388). The threshold value is the midpoint between the two clusters’ centers. **(B)** Scatterplot displaying participants' PLV during AS inside the scanner as a function of the PLV from the SSS-test. Red line represents the correlation of the data. The correlation is displayed for visualization purposes, to emphasize that the synchronization of low synchronizers is consistently worse than that of highs during the AS block. The correlation within groups remains significant only for high synchronizers (r_HIGH_=0.45 p_HIGH_=0.044; r_LOW_=0.21 p_LOW_=0.31). **(C)** Percentage of correct responses for the statistical word-learning task during PL and AS conditions inside the scanner on the entire sample. **(D)** Percentage of correct responses for the statistical word-learning task during PL and AS conditions inside the scanner for the low (blue color) and the high (orange color) synchronizers. The mixed-model analysis of this dataset yielded a significant difference between conditions (main effect of Condition (PL > AS), χ2 = 5.40, *p* < 0.05)), and a main effect of group close to significance (Highs > Lows; χ2 = 3.67, *p* = 0.055), and a trending Condition*Group interaction (χ2 = 2.74, *p* = 0.098). Dots: model predicted group means. Bars: 95% confidence interval. AS: Articulatory Suppression; PL: Passive Listening. Data for S1A and S1B Fig can be found in S5 Data. Data for S1C and S1D Fig can be found in S6 Data.
