# Supplementary material for: Differential activation of a frontoparietal network explains population-level differences in statistical learning from speech
Source: PLoS Biol. 2022 Jul 6;20(7):e3001712. doi: 10.1371/journal.pbio.3001712 (PMC9292101; doi:10.1371/journal.pbio.3001712)
Supplement: S2 Fig — The different networks are shown over a canonical template with MNI coordinates on the upper portion of each slice. Neurological convention is used with a p < 0.05 FWE-corrected threshold at the cluster level and an auxiliary p < 0.001 threshold at the voxel level. In addition to the auditory (green) and frontoparietal (red) networks described in the main manuscript, a sensorimotor (magenta) and a right lateralized fronto-temporo-parietal (yellow) networks were also activated during PL. All these networks were significantly activated during PL for both high and low synchronizers, except the frontoparietal, which was only active for the high. *p < 0.05 Mann–Whitney–Wilcoxon between-group comparison, FDR corrected. Data for S2 Fig (plots) can be found in S7 Data. FDR, false discovery rate; FWE, family-wise error; MNI, Montreal Neurological Institute; PL, passive listening. (DOCX) [file pbio.3001712.s002.docx]

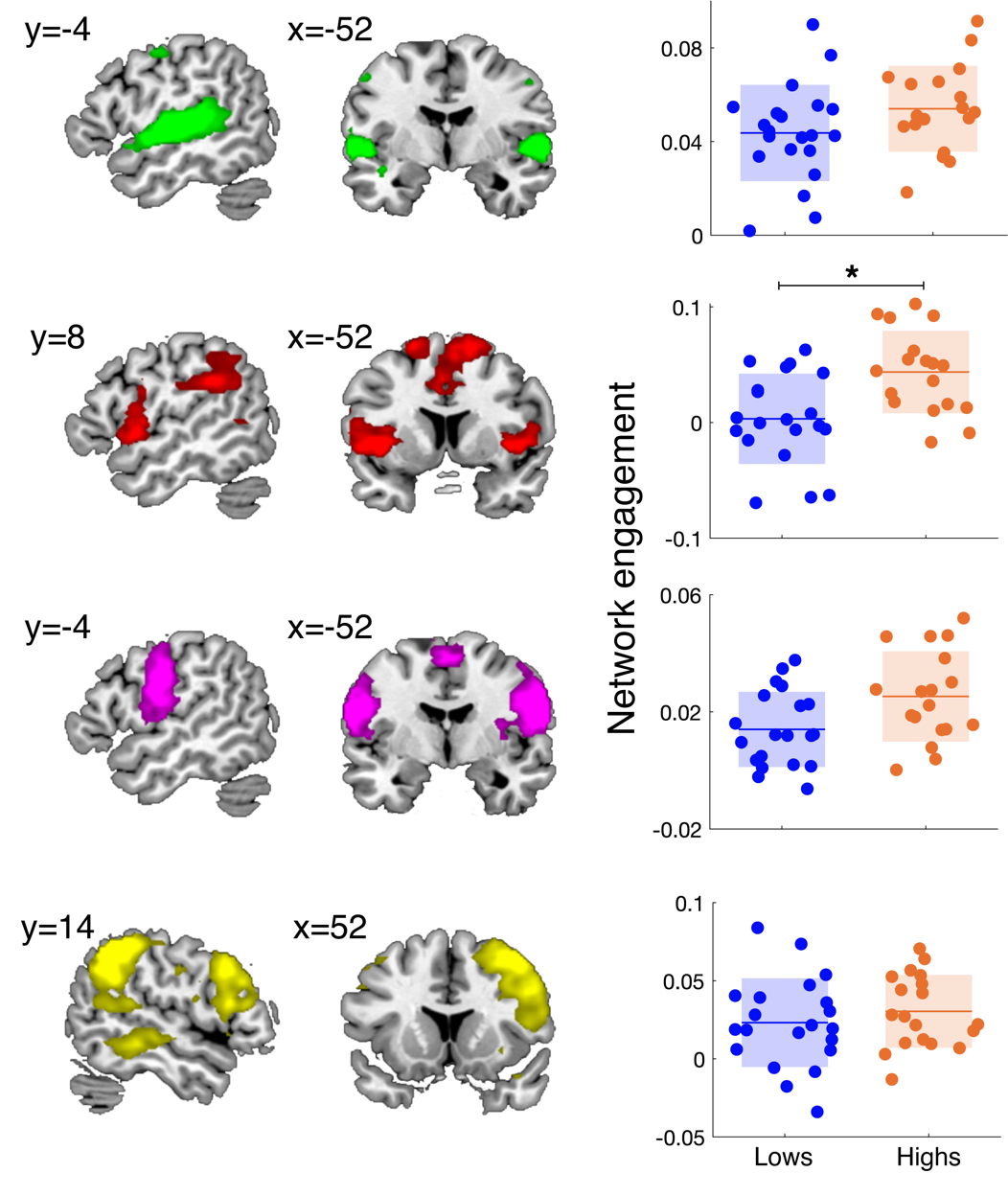


**S2 Fig. Brain networks significantly activated during PL.** The different networks are shown over a canonical template with MNI coordinates on the upper portion of each slice. Neurological convention is used with a *p* < 0.05 FWE-corrected threshold at the cluster level and an auxiliary *p* < 0.001 threshold at the voxel level. In addition to the auditory (green) and fronto-parietal (red) networks described in the main manuscript, a sensorimotor (magenta) and a right lateralized fronto-temporo-parietal (yellow) networks were also activated during PL. All these networks were significantly activated during PL for both high and low synchronizers, except the fronto-parietal, which was only active for the high. **p* < 0.05 Mann-Whitney-Wilcoxon between-group comparison, FDR corrected. Data for S2 Fig (plots) can be found in S7 Data.
