# Supplementary material for: Differential activation of a frontoparietal network explains population-level differences in statistical learning from speech
Source: PLoS Biol. 2022 Jul 6;20(7):e3001712. doi: 10.1371/journal.pbio.3001712 (PMC9292101; doi:10.1371/journal.pbio.3001712)
Supplement: S3 Fig — The different networks are shown over a canonical template with MNI coordinates on the upper portion of each slice. Neurological convention is used with a p < 0.05 FWE-corrected threshold at the cluster level and an auxiliary p < 0.001 threshold at the voxel level. In addition to the auditory (green) and frontoparietal (red) networks described in the main manuscript, a left (light blue) and a right (yellow) lateralized fronto-temporo-parietal network were also activated during AS. All networks were significantly activated during AS for both high and low synchronizers, except for the frontoparietal, which was only active for the high and the auditory, which was marginally significant for the lows (punc = 0.03). Data for S3 Fig (plots) can be found in S3 Data. AS, articulatory suppression; FWE, family-wise error; MNI, Montreal Neurological Institute. (DOCX) [file pbio.3001712.s003.docx]

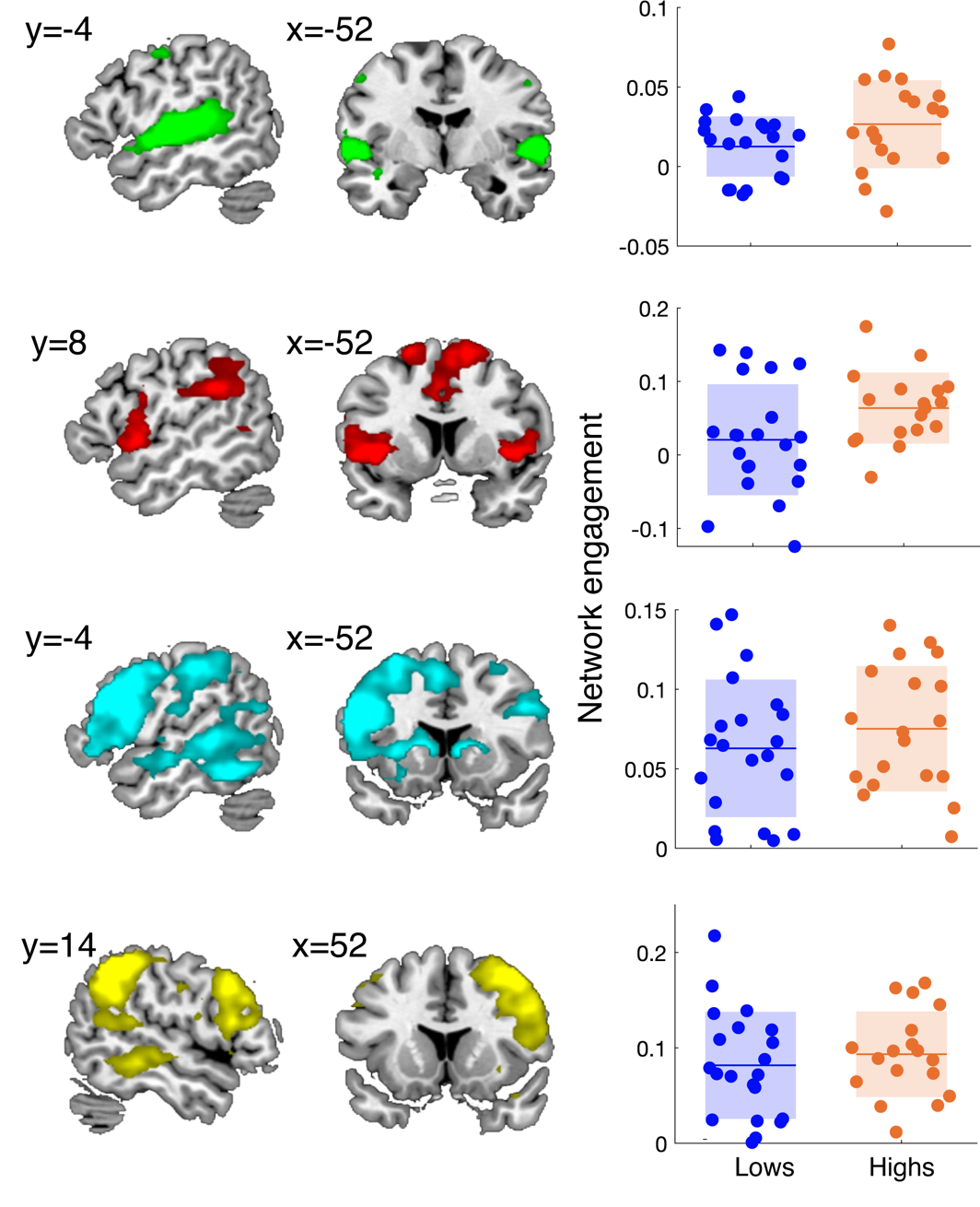


**S3 Fig.** Brain networks significantly activated during AS. The different networks are shown over a canonical template with MNI coordinates on the upper portion of each slice. Neurological convention is used with a *p* < 0.05 FWE-corrected threshold at the cluster level and an auxiliary *p* < 0.001 threshold at the voxel level. In addition to the auditory (green) and fronto-parietal (red) networks described in the main manuscript, a left (light blue) and a right (yellow) lateralized fronto-temporo-parietal network were also activated during AS. All networks were significantly activated during AS for both high and low synchronizers, except for the fronto-parietal which was only active for the high and the auditory, which was marginally significant for the lows (*p*_unc_ = 0.03). Data for S3 Fig (plots) can be found in S3 Data.
