# Supplementary material for: Differential activation of a frontoparietal network explains population-level differences in statistical learning from speech
Source: PLoS Biol. 2022 Jul 6;20(7):e3001712. doi: 10.1371/journal.pbio.3001712 (PMC9292101; doi:10.1371/journal.pbio.3001712)
Supplement: Attachment — Submitted filename: Response_to_Reviewers.docx [file pbio.3001712.s011.docx]

Reviewer #1

This study uses fMRI to investigate individual differences in language-related statistical learning. In a first step, participants were grouped into high and low synchronizers based on a Spontaneous Speech Synchronization test. In a subsequent word learning task, only the high synchronizers activated a fronto-parietal network and showed a behavioural benefit during passive listening compared to an active suppression task.

**We thank the reviewer for the thorough review of the manuscript and for the thoughtful questions that have no doubt helped us enrich the paper. The reviewer will find below our point-by-point answers to these questions which we hope will satisfy his/her concerns.**

1. My main question concerns the group difference between the low and the high synchronizers that also differ in the engagement of the fronto-parietal network. I wonder what the difference between the individuals in the two groups means: Is this a trait difference or simply a state difference, i.e., here most likely a different state of attention while listening?

**We thank the reviewer for raising this important consideration. We cannot, at this point, rule out that group differences relate to different attentional states, as pointed out by the reviewer. We acknowledge this in the manuscript (lines 282 to 285) in mentioning that: ‘*our results line up with recent theories of SL, which postulate the works of both learning systems (e.g., comprising auditory areas in the case of auditory input) and modulatory attentional/control systems (e.g., as supported by fronto-parietal networks) underlying learning performance’.* However, in response to the reviewer’s concern, we have considered it appropriate to add a few lines to the manuscript’s discussion addressing the trait vs state distinction before digging into the possible functional significance of the reported differences. These lines (266 to 272) read:**

***‘Insofar as there are differences between high and low synchronizers at a structural level and differences in synchrony that are stable in time (Assaneo et al., 2019), we understand the high/low synchronizer differences reported in this and previous works as trait differences. We have also theorized on how structural connectivity differences can give rise to the synchrony differences between the groups under particular stimulation conditions (e.g., auditory stimulation within a specific frequency range; Poeppel and Assaneo, 2020). Because of this, our reported group differences could also be understood as state differences, the precise functional significance of which remains an open question.’***

2. How does the fronto-parietal network identified in this study relate to previous work on involvement of the fronto-parietal network in attention and as a hub of cognitive control?

**Thank you for directing our attention to this important literature. We have extended the discussion to further address the potential implication of the fronto-parietal in attention and cognitive control. A new section (lines 288 to 309) now reads:**

***‘On the other hand, there exist multiple and distinct fronto-parietal networks associated with attention that overlap with our reported fronto-parietal network in high synchronizers. Its ventral fronto-parietal component (i.e., inferior prefrontal to inferior parietal cortex), for example, has been related to stimulus-driven attention (Corbetta, Patel, and Shulman, 2008), which may in turn be related to the salience network (Dosenbach et al. 2012). Note that the stimulus-driven attention network is mostly bilateral (e.g., Vossel et al., 2014) but shows different patterns of lateralization, rightward for spatial attention and leftward for temporal attention (Coull et al., 2011). Given the relationship between our fronto-parietal network and auditory-motor synchronization (this paper and Assaneo et al., 2019), a possibility therefore is that high synchronizers’ fronto-parietal engagement relates to a temporal attention mechanism.***

***Another possibility is that fronto-parietal activity in high synchronizers relates to a control network (e.g., Marek and Dosenbach, 2018) that flexibly interacts with other task-specific networks (e.g., Cole et al., 2013; Zanto and Gazzaley, 2013). This is possible given the activity in more dorsal frontal regions that also feature in our reported fronto-parietal network. Interestingly, recent articles (e.g., Albouy et al., 2017; Albouy et al., 2022) show that a supra-modal fronto-parietal network entrains to stimulation (sensory and via rhythmic TMS) in the theta band and that this entrainment (causally) enhances auditory working memory. This is very similar to our previous (Assaneo et al., 2019) and current findings, in which high synchronizers entrain to theta stimulation (higher behavioral PLV and brain-stimulus PLV during passive listening) and show a behavioral advantage over individuals that do not show this entrainment. The extent to which this and the aforementioned fronto-parietal networks are one same network or different networks that interact for high synchronizers during the SL task cannot be answered by our current data and so remains an empirical question. However, our analysis (ICA for fMRI) indicates that, at the very least, these fronto-parietal regions’ time-courses cohere in time.’***

**We believe that this more extended discussion on the potential roles of the fronto-parietal network enriches the paper and hope it satisfies the reviewer’s concern.**

3. Could the authors provide more information about how participants were explicitly instructed in the different tasks? If I understand correctly, there was no task instruction about how to relate their whisper to the heard speech stream. About half of the participants aligned their whisper to the heard stream while the others did not. Would a plausible explanation of the observed results in this study be that participants in the "high" group, i.e. those that engage the fronto-parietal network more, perform a different task than those in the "low" group?

**We apologize for the lack of clarity in the previous version of the manuscript. Indeed, participants were not instructed to synchronize their whispering in either of the tasks (i.e., SSS-test and word learning during AS). We have clarified this in the revised version of the manuscript. The corresponding lines in the Methods section (363 - 372) now read:**

***‘The behavioral protocol consisted of four blocks of statistical word-form learning performed under two different conditions (PL and AS), followed by the SSS-test (see Fig 5A). Four pseudo-languages were generated, and their order was randomized across participants. In the statistical learning blocks, participants were instructed to pay attention to the audio stream to be able to answer post-exposure questions about the perceived sounds. During the PL condition, participants passively listened to two of the pseudo-languages. During AS, participants repeatedly whispered the syllable “tah” while listening to the remaining two pseudo-languages. As in the SSS-test, participants were not instructed to synchronize their speech to the auditory stimulus. Instead, they were told that the point of the whispering was to make the listening task more challenging.’***

**We have also added the reference to our recently published article (Lizcano-Cortés et al., 2022) describing and discussing the synchrony task in detail (line 440).**

**A ‘different task’ hypothesis is hard to reconcile with the instructions given to the participants as well as with the spontaneous nature of the synchrony, its stability across time and tasks, and the fact that it is predictive of brain structural features of the participants. First, synchronization was not instructed in either of the tasks. Participants were simply required to pay attention to the sounds with the purpose of answering a set of questions after exposure. Second, the synchronization between perception and production has been shown to be spontaneous. In a modified version of the SSS-test in which the external syllabic rate is (imperceptibly) increased, high synchronizers still automatically align the produced rate to match the external one (Assaneo et al. 2019, Supplementary Figure 2). Third, the degree of synchrony is a stable feature of the individual. Synchronization values obtained with the SSS-test from the same participant in two sessions completed one month apart are highly correlated (Assaneo et al. 2019, Fig 1). Also, the synchrony obtained during the SSS-test correlates with the one obtained during the AS blocks (this article).**

**According to a ‘different task’ or strategy hypothesis, this would imply that high synchronizers have a stable preference to understand tasks as synchronization assignments. Finally, the SSS-test additionally identifies two groups of participants with different brain structural characteristics (Assaneo et al. 2019, Fig. 3 and Supplementary Figure 8). It would be odd to predict that differences in brain structure are related to different strategies adopted to complete a task. Finally, a ‘different task’ hypothesis is also difficult to reconcile with the fact that a bimodal distribution (high vs low synchronizers) is obtained even when explicit instructions to synchronize are given (see Lizcano-Cortés et al, 2022).**

4. Could the authors elaborate on their interpretation of the correlation between the engagement of the fronto-parietal network with the synchronization (PLV) between the perceived and produced syllables during AS in line 183/184: "suggesting a link between spontaneous auditory-motor synchrony and fronto-parietal network engagement". What does correlation across task performance mean? Is this correlation a marker for attentive participants?

**A few paragraphs (lines 288-322) have now been added to the discussion regarding a possible relationship between the fronto-parietal network and attentional engagement. We hope that this much extended discussion about the potential roles of this network satisfies the reviewer’s concern.**

5. In the second sample three of the behavioural effects were not significant, hence the formulation line 154 "this replication under notably adverse listening/learning conditions (i.e., during fMRI scanning) underscores the robustness of the behavioral results." should be adapted.

**We apologize for the imprecise wording. We meant to highlight the fact that, even inside the scanner, the detrimental effect of AS was restricted to high synchronizers. Following the reviewer’s comment, this section of the results (lines 150 - 156) now reads:**

***“The behavioral performance in the scanner showed the same trend as the learning pattern obtained with the first cohort (Fig S1; Condition (PL > AS): χ2 = 5.40, p < 0.05; Group (Highs > Lows): χ2 = 3.67, p = 0.055; Condition*Group: χ2 = 2.74, p = 0.098; Highs (PL>AS): zratio = 2.32, p < 0.05; Lows (PL vs AS): zratio = 0.08, p = 0.93; Highs vs Lows in PL: zratio = 1.92, p = 0.055; Highs vs Lows in AS: zratio = 0.05, p = 0.96). Even under notably adverse listening/learning conditions (i.e., during fMRI scanning) the detrimental effect of the AS condition was restricted to the high synchronizers.”***

6. Fig 4B: For Subject 2 the engagement of both networks is highly similar, whereas for S2 the networks are rather anti-correlated. The corresponding interpretation from the authors is "This suggests that the learning benefit shown by high synchronizers over lows was driven by a dynamic interplay in time between fronto-parietal and auditory networks." Could you elaborate more on this and make clear why the strong alignment of both networks is interpreted as LESS dynamic interplay?

**We agree with the reviewer that a strong alignment between the networks’ time courses (as shown by S2) could also be interpreted as a strong ’dynamic interplay’ between them. We apologize for the imprecise wording. To amend this, we have modified the corresponding section of the manuscript (lines 209-211) to focus on the fact that the learning benefit shown by high synchronizers is related to the specific pattern of activity highlighted by the XOR rather than a perfect correlation between time courses of the networks. These lines now read:**

***‘This suggests that the learning benefit shown by high synchronizers over lows is related to a specific pattern of activity highlighted by the XOR rather than a perfect correlation between time courses of the networks.’***

7. At the beginning it is stated "Yes - all data are fully available without restriction" and "All relevant data can be found in the paper's Supporting Information files." - However, there are no SI data files and at the end it is stated "DATA AND MATERIALS AVAILABILITY  Correspondence and material requests should be addressed to M. Florencia Assaneo."  This is inconsistent.

**We apologize for the confusion. We removed the DATA AND MATERIALS AVAILABILITY statement from the end of the manuscript. In line with Plos Biology’s guidelines, all relevant data will be made available as SI upon acceptance.**

8. Bar plot in Figures S2 and S3 are not specified - could individual data points be shown (e.g. violin plots) as in the paper it is repeatedly stated how important individual data are.

**We thank the reviewer for pointing out the importance of showing individual data. Following the reviewer’s advice, we modified S2 and S3 accordingly. For consistency with the rest of the plots in the manuscript we plotted the individual data points on the bar plots instead of adopting violin plots.**

9. Scatterplots in Figure 3b show the same data twice - why not color-coding the dots in the left panel for low and high synchronizers and remove the right panel?

Also in the scatterplots shown in Figure 4 it would be informative to colour-code the two groups of synchronizers.

**Following the reviewer’s suggestion, we color coded the two groups in Figure 4. Regarding Figure 3, we added the color code for high and low synchronizers in the left panel of 3b; we would like to keep the right panel, which zooms into the performance of the high synchrony group. In other words, on the one hand, we want to show that if the whole sample is included the correlation remains significant (3b left). On the other, we want to highlight the relationship between the fronto-parietal and AM synch for the high synchronizers, since it is for this group that this network is significantly activated (3b right).**

10. In the scatterplot shown in Fig S1 B: this correlation seems to be driven by the group differences (could you report the within group correlations in addition).

**We thank the reviewer for pointing this out. We have modified the caption of Figure S1B to clarify the purpose of the visualization (lines 771-774). This now reads:**

***“(B) Scatterplot displaying participants' PLV during AS inside the scanner as a function of the PLV from the SSS-test. Red line represents the correlation of the data. The correlation is displayed for visualization purposes, to emphasize that the synchronization of low synchronizers is consistently worse than that of highs during the AS block. The correlation within groups remains significant only for high synchronizers (r_HIGH_=0.45 p_HIGH_=0.044; r_LOW_=0.21 p_LOW_=0.31).”***

Reviewer #2

The authors have previously shown that statistical language learning correlates with participant's auditory-motor synchronization skills, which can be measured with a simple spontaneous speech synchronization (SSS) task. In the new study they replicate and extend this highly interesting finding by using fMRI to investigate the neural bases of individual differences in SSS and statistical learning. The results show that high synchronizers perform better in statistical language learning (replication) than low synchronizers and that they also activate additional fronto-parietal neural network when listening to syllable streams. All participants activated an auditory network. Overall, this is an elegant study that provides new insight into the neural basis of statistical language learning and individual differences, which has been poorly understood. Statistical learning is a fundamental cognitive capacity that is under active investigation among cognitive scientists. I therefore believe that these new findings will be interesting to a wide audience.

**We thank the reviewer for the very positive appraisal of our work and for the many good insightful comments that have no doubt helped to improve it greatly. Below, the reviewer will find a point-by-point response to his/her concerns and how these were addressed.**

1.      Specificity vs. domain-generality. Statistical learning is a domain general learning mechanism, although it is often investigated using speech stimuli. In my opinion the main weakness of the current study is the lack of control conditions/non-speech stimulus streams. The authors link they results tightly to language learning and phonology, although there is there is no direct evidence that the enhanced performance in the SSS task and/or activation of the fronto-parietal network is specific to speech/language. The lack of control stimuli limits the interpretation of the findings and the authors should acknowledge these limitations.

**We thank the reviewer for the important remark. We have now added a paragraph (lines 297-309) in the discussion that comments on the possibility that the fronto-parietal network is in fact a *supra-modal* network as reported in recent literature (e.g., Albouy et al., 2017; Albouy et al., 2022). As in our work, this network has been shown to entrain to sensory stimulation (in their case, visual rotation) in the theta band, which enhances task (in their case, an auditory working memory) performance. Be these networks the same, we agree with the reviewer in that the activation of the fronto-parietal network is not related to language per se even if it affords benefits in language related tasks. This paragraph is inserted within a broader discussion of domain-general networks that show some overlap with our fronto-parietal network (lines 288-322).**

2. Laterality. In their previous paper in Nature Neuroscience the authors reported left-lateralized white-matter and neural entrainment (MEG) differences between high and low synchronizers. In the new study the additional fronto-temporal network seems to be bilateral. This is an intriguing difference between the studies, which would deserve proper discussion.

**We apologize for not addressing these discrepancies between the two studies in the previous version of the manuscript and we thank the reviewer for the opportunity to do so in this revision. We would first like to emphasize that the measures used in both studies are rather different (brain to stimulus PLV and diffusion in Assaneo et al., 2019 vs arbitrary units derived from an ICA of the BOLD signal in the current work). Therefore, a possibility for the discrepancy is that although frontal and parietal regions may work together for synchrony in high synchronizers, as reflected in the ICA for fMRI analysis, each region may perform different computations to achieve that goal, with entrainment in theta occurring only in left frontal regions. That is, other regions within the same network may perform other computations that assist behavioral synchronization that are not captured by the PLV analysis. In terms of the white matter left-lateralization our working hypothesis is that small structural differences can give rise to large functional differences as is the case in high synchronizers (Poeppel and Assaneo, 2020). Again, these are different measures, each revealing potentially different sides of the same phenomenon. We have now made this important point in the discussion, lines 323-332, which read:**

**‘*A possible reason for the lateralization discrepancies with Assaneo et al. (2019) (bilateral engagement vs left lateralization) is the use of radically different measures and analyses (ICA of the BOLD signal vs a phase-locking value between an auditory stimulus and the MEG signals). Thus, although bilateral frontal and parietal regions may work together for synchrony (and learning benefits) in high synchronizers, as reflected in the ICA for fMRI analysis, each region may perform different computations to achieve that goal that are not captured by the PLV analysis, with entrainment in theta occurring only in left frontal regions. We similarly hypothesize that small structural differences (as those reported in Assaneo et al., 2019), as captured by a particular method (diffusion-weighted MRI in that case), can give rise to large functional differences as appears to be the case in high synchronizers (Poeppel and Assaneo, 2020)*.’**

Reviewer #3

In this manuscript, Orpella and colleagues elegantly reconcile apparently disparate neuroimaging findings within the auditory-linguistic statistical learning literature. They build off of prior work demonstrating a link between word segmentation abilities and spontaneous speech auditory-motor synchronization, suggesting that only a subset of learners (high auditory-motor synchronizers) recruit a fronto-parietal network during learning. These same high synchronizers are also most affected when performing a concurrent articulatory suppression task. This manuscript is really strong work—thoroughly motivated, well-written, and the figures were a standout. However, I do feel that its impact could be highlighted to a greater extent, particularly given that a link between high synchronizers and statistical word learning has already been observed. I fully agree that "this work sounds a note of caution about assuming the existence of monolithic mechanisms underlying cognitive tasks" (287-289). Along these lines, the manuscript would be enhanced by more specific, substantial descriptions of how these findings add to the existing literature on individual differences in SL.

**We thank the reviewer for his/her very positive comments about our research and for the thorough revision. Below, the reviewer will find point by point answers to his/her comments and concerns which we hope will be satisfactory.**

MAJOR

1. 274-277: "Broadly speaking, therefore, our results line up with recent theories of SL, which postulate the works of both learning systems (e.g., comprising auditory areas in the case of auditory input) and modulatory attentional/control systems (e.g., as supported by fronto-parietal networks) underlying learning performance."

Given the observed involvement of prefrontal cortex, I recommend richer discussion of how this work relates to proposals that children and adults might recruit different neural learning systems and that it may be worth considering downsides to a mature prefrontal cortex. These very recent refs might be useful:

Smalle et al. (2021). Less is more: Depleting cognitive resources enhances language learning abilities in adults. Journal of Experimental Psychology: General, 150(12), 2423-2434. 
Smalle et al. (2022). Unlocking adults' implicit statistical learning by cognitive depletion. PNAS, 119 (2), e2026011119.

**We thank the reviewer for pointing out this apparent discrepancy between our results and these very interesting works by Smalle et al. We have addressed this in a new paragraph in the discussion (lines 310-322) that reads:**

***‘There are also reasons to distinguish these fronto-parietal networks from the dorsal network for goal-directed attention (Corbetta and Shulman, 2002), despite a similar involvement of dorsal prefrontal regions. In contrast to research showing SL benefits from interfering with this network (e.g., Smalle et al., 2021; 2022), we show that articulatory suppression hinders learning. Moreover, fronto-parietal involvement, which correlates with auditory-motor synchronization, confers a learning benefit during passive listening. It is therefore likely that the reported dorsal prefrontal regions, which are shown to cohere in time with other fronto-parietal regions, perform a role different from goal-oriented attention within the context of our tasks. This is in line with the idea that the same region can undertake different roles depending on its interactions with other regions. It was not possible to determine the precise role of prefrontal regions alone from our data. On the other hand, we also show that the learning benefit relates to the way the fronto-parietal network interacts with the auditory network. Another possibility, therefore, is that different kinds of dorsal prefrontal involvement during learning incur in either learning benefits or hindrance.’***

2. Fig. 4; 186-210: The incorporation of behavioral data into the neuro analysis is a strength of this paper. However, it is worth noting that there is a substantial push in the SL literature towards more effectively capturing individual differences in SL.

Siegelman, N., Bogaerts, L., & Frost, R. (2017). Measuring individual differences in statistical learning: Current pitfalls and possible solutions. Behavior Research Methods, 49(2), 418-432.

The present work involves tests of SL with few trials, test items of equivalent difficulty, and, in the case of the learning benefit analysis, difference scores (see discussion in Trafimow (2015)—A defense against the alleged unreliability of difference scores). Can the authors discuss/ demonstrate the reliability of the behavioral measures used?

**We thank the reviewer for raising this important issue in the SL literature. We would first like to emphasize that, while acknowledging the need for more nuanced measures that capture not only individual differences in overall SL but also in the time course of learning, the focus of the current paper is to trace group-level differences based on what we think is an interesting robust dividing measure (auditory-motor synchrony). In this sense, our work speaks of individual differences. At this level, we have replicated the learning differences between the two groups (highs and lows) on several occasions now, an important datum that we have now emphasized in the new version of the manuscript (lines 129-131) as:**

**‘*The analysis also showed a main effect of Group (Highs > Lows; χ2 = 9.11, p < 0.01) in line with our previous work (Assaneo et al., 2019) …*’**

**We have also placed more emphasis on individual differences research on SL in lines 340-343:**

***‘This work, therefore, not only highlights the importance of considering individual differences in SL (Siegelman, Bogaerts, and Frost, 2017) but also sounds a note of caution about assuming the existence of monolithic mechanisms underlying such cognitive tasks.*’**

MINOR

3. 313-315: "It is assumed that the executive load imposed by the repeated syllable articulation in this paradigm is minimal, given the highly automatized nature of the articulation subtask." Can the authors provide a stronger justification for this?

**We apologize for the lack of clarity in the previous version of the manuscript. We have reworded the corresponding lines (372-375) in the Methods section to:**

**‘*In line with the previous literature (Baddeley et al. 1984; López-Barroso et al. 2011), we assumed that any effects of AS on statistical learning would be due to an interference with the articulatory loop rather than to a higher executive load, which would be very unlikely given the highly automatized nature of the articulation subtask*.’**

4.  410-450: It's not clear me whether the test phase was included when convolving the timing of the AS and PL conditions.

**We thank the reviewer for pointing out the need to specify this information. We have added the following lines to the manuscript (lines 500-503):**

***‘The test phase was also included in the GLM model as a separate nuisance condition. Therefore, the beta values for the AS and PL conditions were computed from the part of the fMRI signal pertaining to the listening blocks (i.e., without the testing phase, which was modeled separately).’***
